# New Insights into the Developing Rabbit Brain Using Diffusion Tensor Tractography and Generalized q-Sampling MRI

**DOI:** 10.1371/journal.pone.0119932

**Published:** 2015-03-23

**Authors:** Seong Yong Lim, Yeu-Sheng Tyan, Yi-Ping Chao, Fang-Yu Nien, Jun-Cheng Weng

**Affiliations:** 1 School of Medical Imaging and Radiological Sciences, Chung Shan Medical University, Taichung, Taiwan; 2 Department of Medical Imaging, Chung Shan Medical University Hospital, Taichung, Taiwan; 3 School of Medicine, Chung Shan Medical University, Taichung, Taiwan; 4 Department of Computer Science and Information Engineering, Graduate Institute of Medical Mechatronics, Chang-Gung University, Taoyuan, Taiwan; National Yang-Ming University, TAIWAN

## Abstract

The use of modern neuroimaging methods to characterize the complex anatomy of brain development at different stages reveals an enormous wealth of information in understanding this highly ordered process and provides clues to detect neurological and neurobehavioral disorders that have their origin in early structural and functional cerebral maturation. Non-invasive diffusion tensor magnetic resonance imaging (DTI) is able to distinguish cerebral microscopic structures, especially in the white matter regions. However, DTI is unable to resolve the complicated neural structure, i.e., the fiber crossing that is frequently observed during the maturation process. To overcome this limitation, several methods have been proposed. One such method, generalized q-sampling imaging (GQI), can be applied to a variety of datasets, including the single shell, multi-shell or grid sampling schemes that are believed to be able to resolve the complicated crossing fibers. Rabbits have been widely used for neurodevelopment research because they exhibit human-like timing of perinatal brain white matter maturation. Here, we present a longitudinal study using both DTI and GQI to demonstrate the changes in cerebral maturation of in vivo developing rabbit brains over a period of 40 weeks. Fractional anisotropy (FA) of DTI and generalized fractional anisotropy (GFA) of GQI indices demonstrated that the white matter anisotropy increased with age, with GFA exhibiting an increase in the hippocampus as well. Normalized quantitative anisotropy (NQA) of GQI also revealed an increase in the hippocampus, allowing us to observe the changes in gray matter as well. Regional and whole brain DTI tractography also demonstrated refinement in fiber pathway architecture with maturation. We concluded that DTI and GQI results were able to characterize the white matter anisotropy changes, whereas GQI provided further information about the gray matter hippocampus area. This developing rabbit brain DTI and GQI database could also be used for educational purposes and neuroscience investigations.

## Introduction

Understanding the development and senescence of the brain systems that are associated with profound alterations in connective anatomy is of great clinical importance because most neurological and neurobehavioral disorders have their origin in early structural and functional cerebral maturation. Animal models have been essential for the understanding of the brain and neurodevelopment. Several species have been used in neuroscience research, from primates to small animals such as rat, mouse and rabbit. Rabbits have many advantages over other animal species; principally, rabbits exhibit human-like timing of perinatal brain white matter maturation and have been widely employed to model brain damage after perinatal injury in humans [1]. Rabbit models of cerebral palsy [1, 2], intrauterine inflammation [3], and intrauterine growth restriction [4] have been developed, demonstrating changes in neonatal neurobehavioral and in brain structure [5].

By using conventional MRI, we have been able to macroscopically delineate early development events such as myelination and gyral development. Studies by Barkovich et al. demonstrated changes in signal intensity on T1- and T2-weighted images during brain maturation of children and these have been attributed to decreases in brain water content and the presence of myelination process [6]. Diffusion tensor magnetic resonance imaging (DTI) is another MR modality that can microscopically reveal water diffusion in the biological tissue [7], allowing the alterations in brain water diffusion caused by the cerebral maturational processes in neonatal brain to be analyzed quantitatively. The values of the water diffusion parameters differ markedly between neonate/pediatric brain and adult brain, and these parameters vary with age. DTI studies by Huppi et al. [8], Neil et al. [9], Mukherjee et al. [10] have shown that water molecule mean diffusivity decreases with age in both gray and white matter, diffusion anisotropy increases with age especially in white matter region. Brain water content; the formation of new barriers to water mobility, such as cell membranes associated with the outgrowth of axons and dendrites; and white matter myelination, are regarded as factors that would affect the water molecule mean diffusivity.

Water diffusion maps derived from DTI may provide the means for the early detection of irreversible brain injury, which is particularly critical in the context of the administration of therapies to neonates [11]. With the development of 3D diffusion tensor fiber tractography, cerebral maturation, especially of the white matter pathway, and its consequent connectivity can be followed throughout neonate development into adulthood, with the potential to study the correlations between the abnormalities found on DTI and the ultimate neurological or neurobehavioral outcomes [12]. It is worth noting that the precise DTI water diffusion parameters that are to be employed are still open to debate. The principal orientations, which are the eigenvectors that were obtained from the diffusion tensors, have allowed the tracking of the axonal fiber tracts and have revealed the white matter connections between cortical areas. However, due to the inherent limitation of the Gaussian tensor model, DTI cannot resolve complicated structures, such as the crossing or branching neural fibers [13, 14]. Furthermore, the white matter maturational myelination process would significantly affect the diffusion anisotropy as well.

To overcome these problems and to better characterize the complicated fiber structures, several reconstruction methods combined with proper acquisition and diffusion encoding have been proposed. These reconstruction methods typically have their own applicable diffusion sampling schemes, which may include a single-shell scheme used by high angular resolution diffusion imaging (HARDI) [15]; a multiple-shell scheme [16, 17]; or a grid scheme used by diffusion spectrum imaging (DSI) [18]. The model-free approaches or the q-space imaging methods are based on the Fourier relationship between the diffusion MR signals and the underlying diffusion propagator [19]. Based on the data acquired through the HARDI scheme, Tuch proposed q-ball imaging (QBI) to estimate the diffusion ODF of the diffusion propagators using the Funk-Radon transformation [20]. By using a grid sampling scheme, Wedeen proposed diffusion spectrum imaging (DSI), which calculates ODF by applying the Fourier transform to the diffusion MR signals. To further extend the applicability of q-space imaging, Yeh et al. proposed generalized q-sampling imaging (GQI), which can be applied to a variety of datasets under a balanced sampling scheme such as the single-shell, multi-shell or grid sampling schemes, with accuracy comparable to QBI and DSI. Furthermore, GQI is believed to be able to resolve complicated crossing fibers [21].

Studies by D'Arceuil et al. using fixed rabbit brains to observe the developmental changes in regional diffusion anisotropy and white matter fiber tract development have demonstrated that white matter anisotropy increases with age [22]. To provide a unique insight into the structural basis of cerebral maturation as well as to characterize developmental changes more accurately for future injury or disease investigations, we proposed the usage of both DTI and GQI to demonstrate the longitudinal changes of the in vivo developing rabbit brain.

## Materials and Methods

### Animal preparation

All the procedures of the animal experiment adhered to the Guidelines for Care and Use of Experimental Animals by the Laboratory Animal Center at the Chung Shan Medical University, Taichung, Taiwan. The protocol was approved by the Committee on the Ethics of Animal Experiments of the Chung Shan Medical University, Taichung, Taiwan (Permit Number: 1219). Five adult male New Zealand white rabbits (aged from 4 weeks to 40 weeks) were used in this longitudinal experiment. The rabbits were singly housed in a climate-controlled environment on a 12-hour light-dark cycle with food and water available ad libitum.

For the MRI scan, each experimental animal was placed in the prone position and was anesthetized using an inhalation anesthesia mix of isoflurane (5% induction and 2.5% maintenance) and oxygen (300 mL/min) via a homemade plastic nasal/oral mask, and all efforts were made to minimize suffering. A real-time visual monitoring system was used to confirm the immobilization of the animals during the MRI scan. The heart rate and respiratory rate were monitored throughout the scan, and the temperature (37°C) was maintained via circulating heat pads.

### Data acquisition

In our study, a brain MRI was performed on all five rabbits every four weeks, starting from the 4^th^ week until the 24^th^ week as well as on the 32^nd^ and 40^th^ week (8 time points in total) using a 1.5 Tesla MR scanner (Magnetom Sonata, Siemens Medical Solutions, Erlangen, Germany) with double loop array coils.

Whole-brain coronal T2-weighted images (T2WIs) were obtained using a turbo spin echo with the following parameters: repetition time (TR)/echo time (TE) = 4330 ms/114 ms, resolution = 0.39 x 0.78 mm^2^, slice thickness = 1.5 mm, number of excitations (NEX) = 13, number of slices = 30 and scan time = 9 min 33 sec. To improve the detection sensitivity over the full extent of T2 changes caused by the maturation of rabbit brain, image data for R2 mapping were acquired. To obtain R2 mapping, single-slice multi-echo spin echo sequence with half spatial resolution was performed to acquire 32 sets of images corresponding to 32 different TEs, ranging from 15 to 480 ms, to sample along the decay of transverse magnetization, with the following parameters: TR = 2000 ms, slice thickness = 2 mm, NEX = 4, and scan time = 8 min 38 sec.

The diffusion data were acquired using a multi-slice, single-shot spin echo- echo planar imaging (EPI) sequence with TR/TE = 2900 ms/133 ms, resolution = 0.78 x 0.78 mm^2^, slice thickness = 2 mm, NEX = 9, and number of slices = 12. Coronal scanning was carried out contiguously from the olfactory bulb to the end of the cerebrum (done in two parts). We used 12 directions of the diffusion gradient, and each encoding direction was composed of 8 increments corresponding to b values ranging incrementally from 0 to 2000 s/mm^2^ (with a 250 s/mm^2^ interval). The total scan time for the diffusion acquisition was approximately 42 min for each part.

### Image analysis

For T2WI and R2 mapping, the changes of the regional brain size and myelination were evaluated. We calculated the signal intensity in both sides of the brain regions in the T2WI, including the corpus callosum, hippocampus, olfactory tract and cerebral cortex. The size of each brain region was divided by the size of whole brain. All of the results are expressed as the mean ± standard error (SE), and statistical analysis was performed and compared across the development of rabbit brain. In the R2 mapping, we calculated the changes of the R2 value in the hippocampus and corpus callosum, which could imply the myelination status.

Image reconstruction of DTI and GQI was performed using DSI Studio (National Taiwan University, Taiwan). The GQI reconstruction was implemented with a diffusion sampling ratio of 1.3; for the majority of the application, we are observing restricted diffusion, and a ratio 1.3 should covers most of the diffusion, as suggested in the GQI study [21]. For tractography, four structures (cortex, hippocampus, corpus callosum, and olfactory tract) were selected for the tracking of their fiber pathway, and the whole brain tractography was also demonstrated.

For the diffusion tensor imaging (DTI) analysis, the fractional anisotropy (FA), mean diffusivity (MD), axial diffusivity (AD) and radial diffusivity (RD) mapping were calculated. For the generalized q-sampling imaging (GQI) analysis, the normalized quantitative anisotropy (NQA), generalized fractional anisotropy (GFA), and isotropic value of the ODF (ISO) were calculated. The precise definition of these new diffusion indices from GQI can be found on the website of DSI studio (http://dsi-studio.labsolver.org/Manual/Reconstruction) or on GQI paper [21]. The ROIs of the corpus callosum, bilateral olfactory bulbs, hippocampi and cerebral cortex were manually drawn by an expert operator on four consecutive slices on GFA maps. Same ROIs were then used to calculate changes of FA, MD, AD, RD, NQA and ISO across our longitudinal study. All results are expressed as the mean ± SE, and the normalized ratios (the value of ROI divided by the value of cortex) were calculated for statistical analysis and compared across the development and maturation of rabbit.

For the statistical analysis, a paired t-test was used to detect any significant differences between the maturation time points in each of the 8 examined compartments of the diffusion (DTI and GQI) indices and the anatomical images (T2WI, R2 mapping). A p-value of <0.05 was considered statistically significant.

## Results

### Regional brain size and R2 mapping

**Fig 1:**
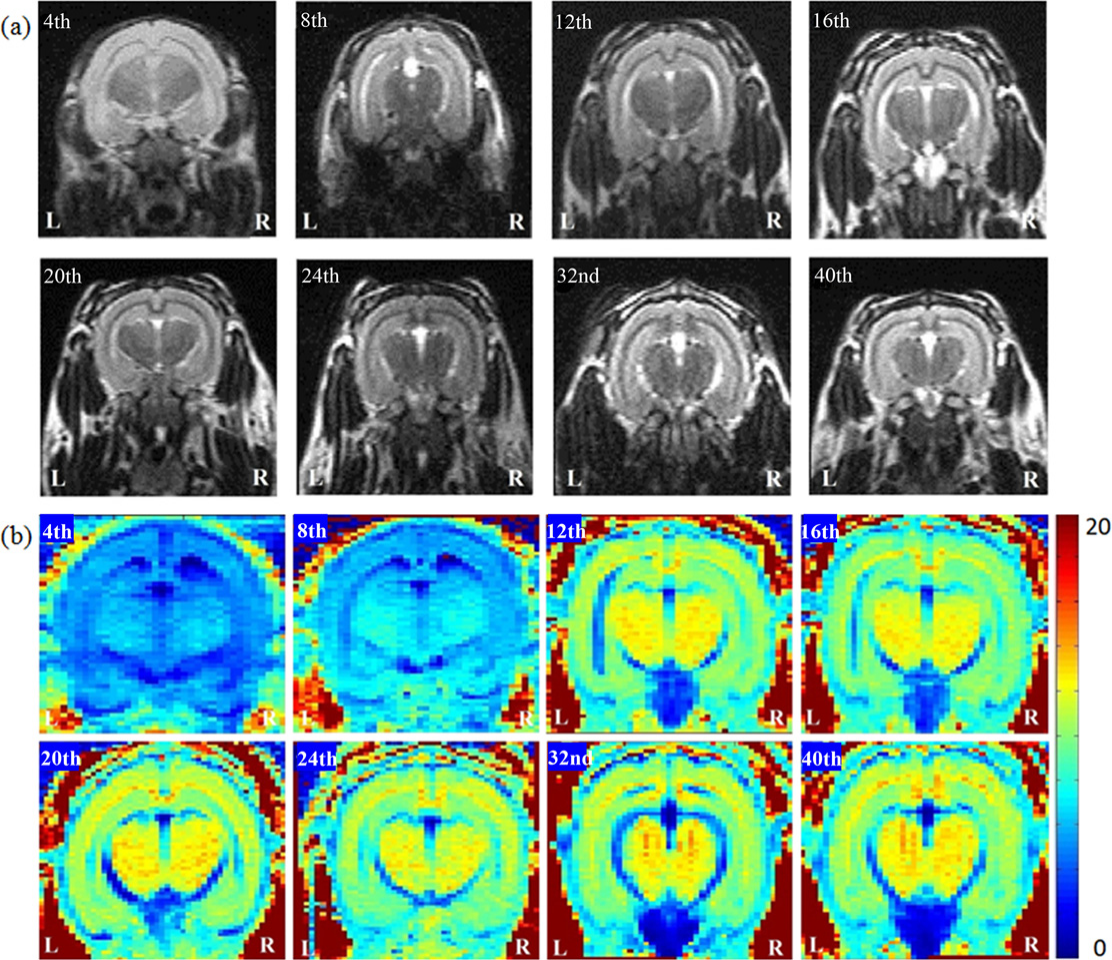
The anatomical MR images, (a) T2W images and (b) R2 mapping, in 4- to 40-week-old developing rabbit brains. Using R2 mapping, we observed that the intensity in the regions of corpus callosum and hippocampus increased through week 12 prominently.

**Fig 2:**
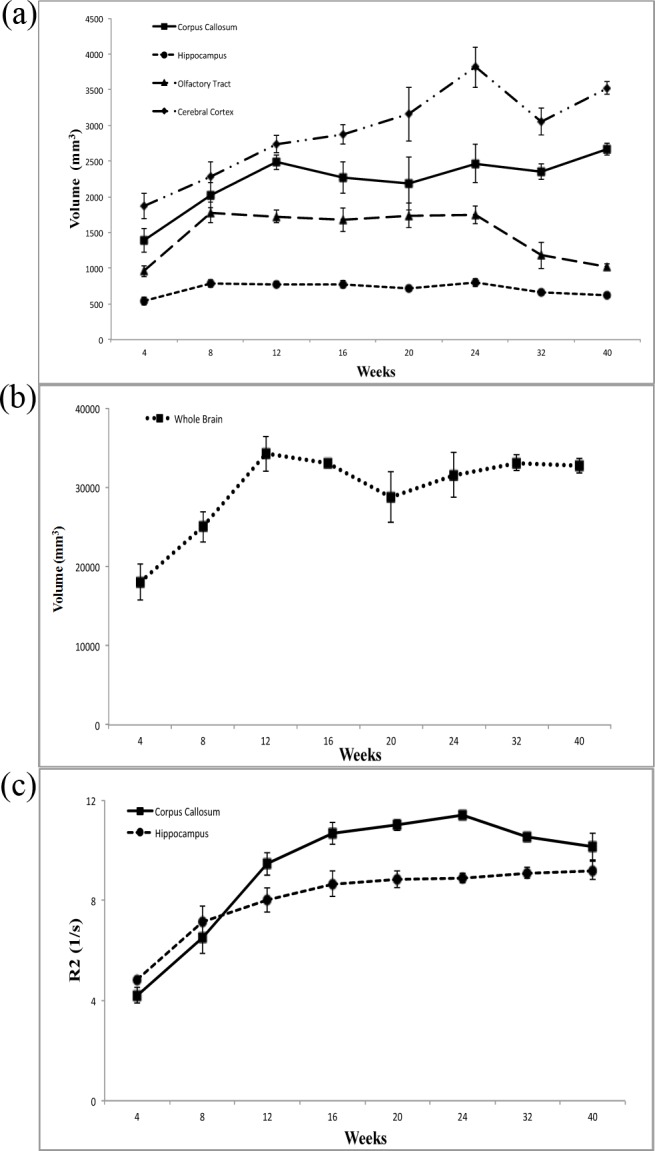
The changes in (a) regional brain size and (b) whole brain size in 4- to 40-week-old developing rabbit brains. (c) The changes in the R2 values of the corpus callosum and hippocampus in developing rabbit brains from 4 to 40 weeks.

In Figs. 1 and 2, by measuring the regional volume of the rabbit brain, we observed a continuous increase in the cerebral cortex after the 4^th^ week. With respect to others structures, including the olfactory tract and hippocampus, we observed an increase from the 4^th^ to 8^th^ week, although there were no observable changes during subsequent weeks. In the corpus callosum, the regional volume increased from the 4^th^ to 12^th^ week before reaching to a plateau until the 20^th^ week; later, there was an observable increase on 24^th^ week. With respect to the observation of the volume of whole brain, there was a very prominent increase from week 4 to week 12 before reaching to a plateau on subsequent weeks.

Using R2 mapping, we observed that the intensity in the regions of corpus callosum and hippocampus increased prominently through week 12, with values of R2 increasing from 4.19±0.32 /sec to 9.45±0.44 /sec for corpus callosum; and from 4.83±0.08 /sec to 8.02±0.49 /sec for hippocampus. In Fig. 2c, the prominent continuous increase in the values of R2 for both the corpus callosum and hippocampus in the developing rabbit brains from 4 to 24 weeks can be observed.

### Tractography

**Fig 3:**
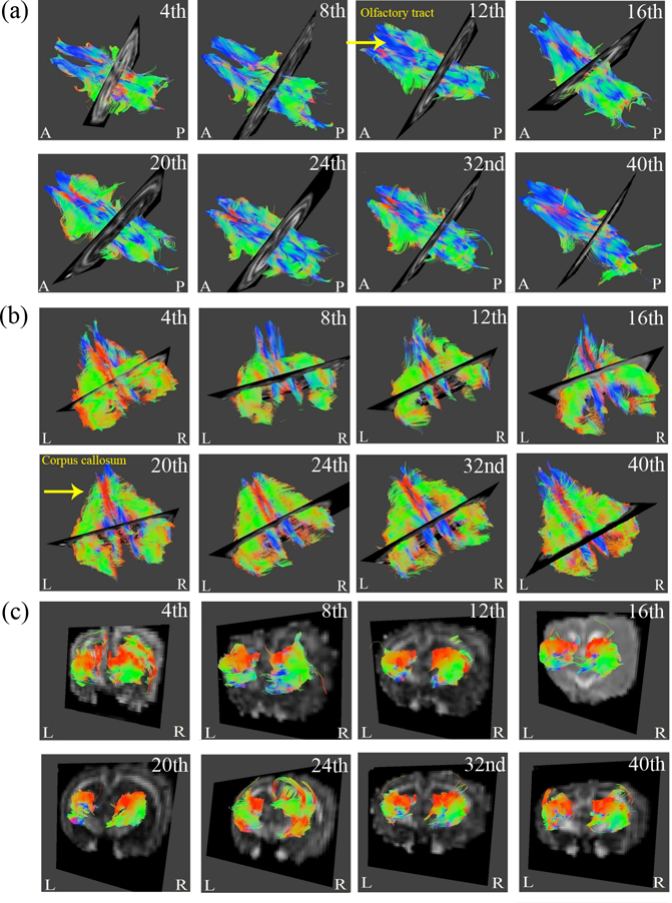
Regional brain DTI tractography from 4- to 40-week-old rabbits. The three regions presented in (a) to (c) include the olfactory tracts, corpus callosum and hippocampus, respectively. (L: Left; R: Right; A: Anterior; P: Posterior.)

**Fig 4:**
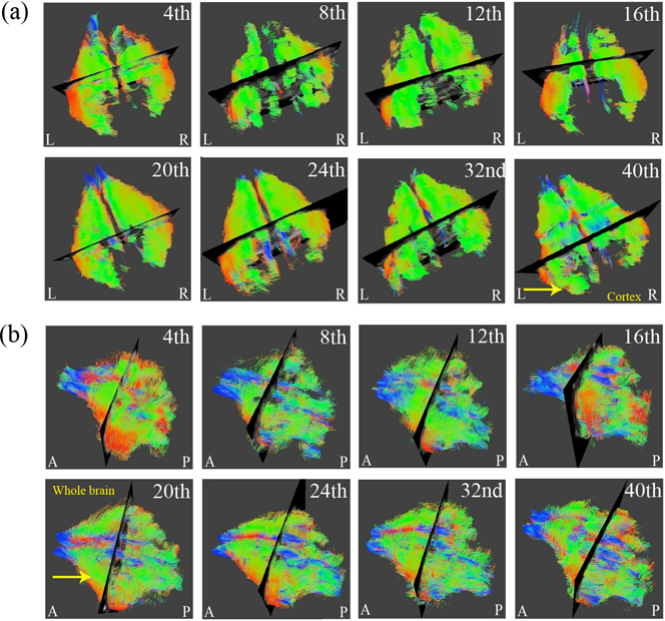
The DTI tractography of (a) cerebral cortex and (b) whole brain from 4- to 40-week-old rabbits (L: Left; R: Right; A: Anterior; P: Posterior).

In Fig. 3, the rendered fiber pathway of the major white matter tracts, such as the corpus callosum and olfactory tract, as well as the gray matter, such as the hippocampus, exhibited refinement in the regional tract architecture with increasing age. Tracking the results of the cerebral cortex and whole brain from Fig. 4 also validated the cerebral maturation as the complex and complicated fibers were observed to be more compact and well organized.

### DTI indices

**Fig 5:**
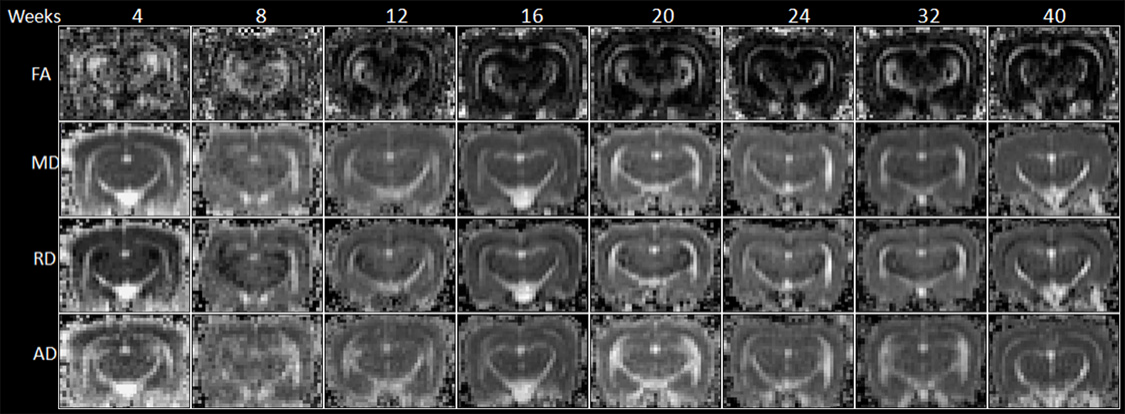
The FA, MD, RD and AD mapping of 4- to 40-week-old rabbit brains derived from DTI.

**Fig 6:**
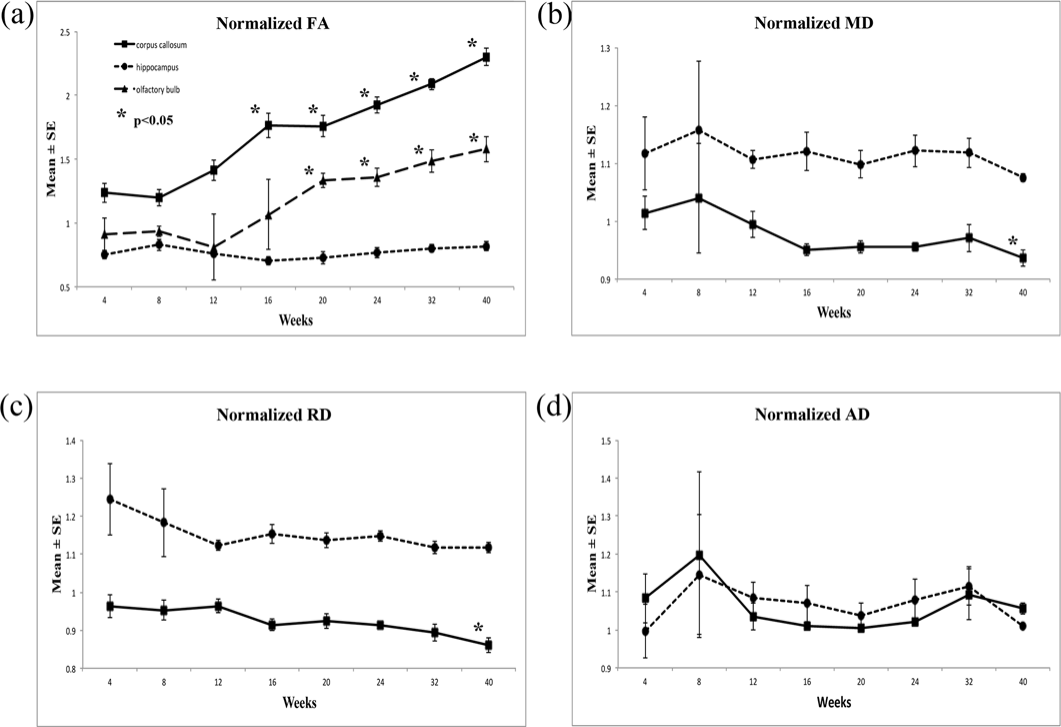
The longitudinal changes in diffusion indices in DTI, including FA, MD, RD and AD, with respect to the structures of corpus callous, hippocampus and olfactory bulb of 4- to 40-week-old rabbit brains.

As shown in Figs. 5 and 6, the normalized FA value of corpus callosum and olfactory bulb increased with age, and there was no significant change in the normalized FA of the hippocampus with age. The value of normalized FA for the corpus callosum, the major white matter tract, first dip in the 4^th^ to 8^th^ week, yet the subsequent increase in the following weeks was very prominent. For olfactory bulb, there was a small decrease from the 8^th^ to 12^th^ week, yet in the subsequent weeks, the increase was equally prominent. The normalized MD and normalized RD of the corpus callosum and hippocampus decreased with age, and there was no significant change in the normalized AD of these structures with age.

### GQI indices

**Fig 7:**
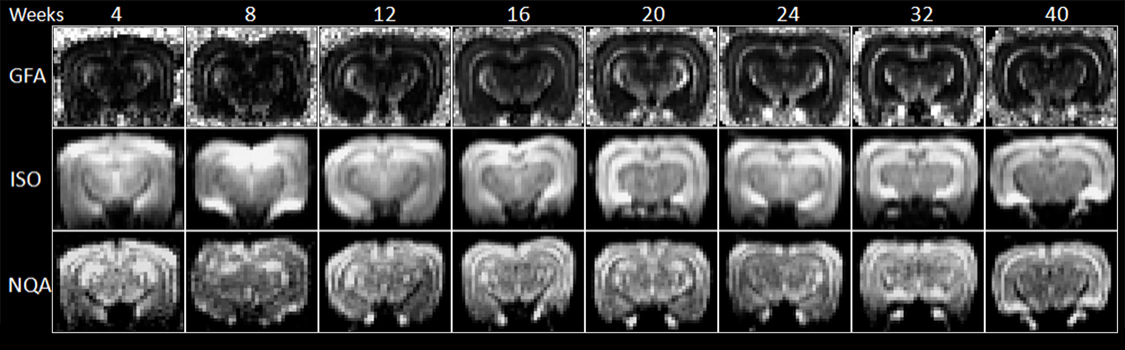
GFA, ISO and NQA mapping of 4- to 40-week-old rabbit brains derived from GQI.

**Fig 8:**
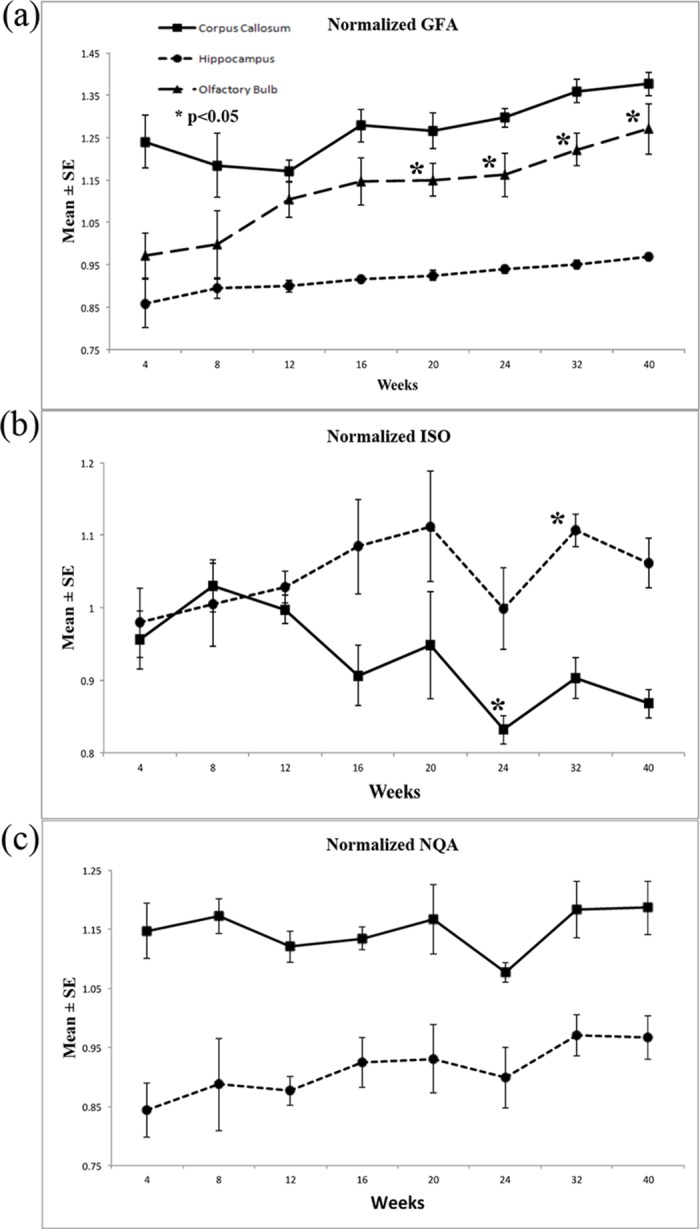
The longitudinal changes in diffusion indices in GQI, including GFA, ISO and NQA, with respect to the structures of corpus callous, hippocampus and olfactory bulb of 4- to 40-week-old rabbit brains.

In Figs. 7 and 8, we observed that the normalized GFA of the corpus callosum, hippocampus and olfactory bulb increased with age. The normalized GFA value of the structure of the corpus callosum, the major white matter tract, first dip in the 4^th^ to 12^th^ week, although the subsequent increase in the following weeks was very prominent. With respect to the structures of the olfactory bulb and hippocampus, there was a continuous significant increase beginning in the 4^th^ week. Overall, the normalized ISO value of the corpus callosum decreased with age and increased in the structure of the hippocampus. With respect to the value of normalized NQA, changes in the hippocampus exhibited a more prominent increase as the brain developed.

## Discussion

### R2 mapping

According to the results of R2 mapping (Fig. 1b), by the 12^th^ week, the intensity of the whole brain increased prominently, especially in the regions of the corpus callosum and hippocampus as the brain developed. Fig. 2c demonstrates that the calculated R2 values of the corpus callosum and hippocampus gradually increased with age. D'Arceuil et al. reported that the myelination process of the rabbit brain corpus callosum persisted through at least the 4^th^ week after birth [22]. Weng et al. observed the thickening of the myelin sheath of the corpus callosum of rat from the 3^rd^ week to 3^rd^ month [23]. Studies by Fukunaga et al. also reported that the presence of myelin or myelination process may have caused the R2 value to increase [24]. By using R2 mapping, we observed that the increasing R2 values is consistent with the increase of myelination process during the same period of cerebral maturation, which may further confirm myelination relationship with the water diffusion parameters.

### Tractography

Tractography, commonly known as fiber tracking, is another technique that can be applied to the developing brain to quantitatively assess specific fiber pathway maturation, especially in the white matter regions. Berman et al. were able to demonstrate significant differences in the maturational changes in FA between the motor and somatosensory pathway in premature infants, further validating this approach for use in measuring the diffusion changes across multiple different levels of functional tracts [25].

In Figs. 3 and 4, the colors of red, green and blue represented fibers in the directions of superior-inferior, left-right and anterior-posterior. Fig. 3a showed prominent increases of blue fibers along the tract especially after 12^th^ week inferred the maturational refinements in the anterior-posterior pathway of olfactory tracts. Fig. 3b showed prominent increases of green fibers as early as after 20^th^ week that inferred the maturational refinements in the direction of left to right, which is the major fiber pathway of corpus callosum that have contralateral axonal projections for connecting the left and right cerebral hemispheres. As for the results of the cerebral cortex in Fig. 4a, more abundant fibers of all orientations could be observed, especially after 40^th^ week which may infer that the fibers of gray matter structure grew in all different directions. Finally for the tracking of whole brain from Fig. 4b, it is apparent that a more compact and well organized fiber pathway of all directions had been developed especially after 20^th^ week.

### Diffusion indices

As we mentioned earlier, the diffusion indices or anisotropy values differ markedly between pediatric and adult brain [26]. With respect to white matter areas, the diffusion indices are relatively low for newborns and increase steadily with the increasing age [27]. We could observe the same trend in the normalized FA values of DTI and normalized GFA values of GQI for the corpus callosum as well. The definition of GFA is documented in the Q-ball Imaging paper and it is calculated from an orientation distribution function (ODF), note that like the FA for DTI, GFA is automatically normalized to [0, 1] as well. According to the paper, scalar measures on the ODF are useful to define tissue contrast, perform statistical analyses, or summarize geometric properties of the ODF [20].

According to Tuch QBI paper [20], DTI’s inability to resolve intravoxel orientational heterogeneity stems from the limitation of tensor model itself, which assumes a single Gaussian diffusion compartment within each voxel. The Gaussian function has only a single directional maximum and therefore cannot adequately describe diffusion functions with multiple maxima. The fiber crossing confound in DTI has prompted efforts to develop methods capable of resolving intravoxel fiber crossing, such as HARDI, QBI, DSI, GQI [13–18, 20, 21].

For the trend of normalized FA change in Fig. 6a and normalized GFA in Fig. 8a which looked similar but different at the first 3 time points, we believed that these may be resulted from the presence of crossing fibers in the corpus callosum which cannot be resolved from DTI. Myelination process should have taken place as well, yet we speculated that maybe the effect on diffusion anisotropy may not be adequate to cause changes yet. Subsequently after these 3 time points, myelination process may have taken place more rigorously which provided similar increasing trend for both normalized FA and normalized GFA. From these observations, we could infer from the trend difference at the first 3 time points that GQI diffusion indices are the more appropriate representations for the underlying changes caused by the crossing fibers.

Yeh et al. introduced an index known as quantitative anisotropy (QA) for GQI to quantify the spin population in a specific direction which is believed to be able to resolve complicated crossing fibers, especially in the regions of gray matter [21]. The normalized QA (NQA) scales the maximum QA value of a subject to 1 so that QA may be more comparable across subject. NQA values introduced in GQI demonstrated a prominent increase in the gray matter structure of the hippocampus, giving us the opportunity to observe gray matter changes during the maturational process.

According to technical review on DTI of normal and injured developing human brain [28], the precise contribution to the decrease in the values of MD with increasing age is not known, although it has been postulated that the rapid decrease observed between early gestation and term is due to the concomitant decrease in overall water content [9]. As it does, structures that hinder water motion (e.g. cell and axonal membranes) become more densely packed, increasing restriction to motion; as if the brain becomes more viscous as its water content decreases. During rabbit brain maturation, our normalized MD values decrease with increasing age, as observed in the structures of the corpus callosum and hippocampus. The same trend could be observed in the values of normalized ISO in the structures of the corpus callosum. However, a different increasing trend could be observed in the hippocampus. ISO used the minimum value of an ODF as the background isotropic diffusion component, as documented in Yeh’s 2010 GQI paper [21]; again another GQI index give us the opportunity to observe gray matter changes during the maturational process.

Through the measurements of three eigenvectors, we may infer that not all the changes in MD are due to the reduction in the overall brain water content. Water diffusion in the perpendicular direction to white matter fibers, which is observed principally in RD, decreased with increasing age, as demonstrated prominently by our results. This result may indicate changes due to premyelination (change of axonal width) and myelination [29]. As the anisotropy values of the cortical gray matter in rabbit brain after birth are generally consistent; this fact represents the main reason we chose to use cortex anisotropy values for normalization.

### Comparison between GQI and DTI indices

The microstructural restriction in white matter due to myelin sheath, axonal packing and axonal membranes increases with age, leading to greater non-Gaussian diffusion and non-monoexponential b-value dependence. Hence, approaches that are more accurate and sophisticated than conventional DTI are needed to extract extra information regarding such restricted diffusion environments. GQI describes the water diffusion behavior by the Fourier relationship between water diffusion and signal decay. The use of this relationship ensures more sensitive characterization than fitting the signal decay to a monoexponential model. As a model-free method, GQI overcomes the limitation of DTI, which was unable to resolve the complicated neural structures, i.e., fiber crossing. Therefore, GQI-derived diffusion indices can greatly improve the level of significance and specificity in the analysis along axial direction.

Previous study demonstrated that various diffusion indices estimated by conventional DTI, including FA, are b-value-dependent. While applying different b-values may provide different physiological information, comparison among conventional DTI studies must be made with caution [30]. The assumption of the monoexponential attenuation due to diffusion is invalid when the b-value is high, but high b-values can probe high diffusion frequency information. The GQI demonstrated in the present study improves the detectability of the microstructural changes during brain maturation.

### Limitations

Conventional DTI protocol required at least one b-value with 6 independent diffusion gradient directions and one null image, while higher order HARDI protocol required more diffusion gradient directions and higher b-values. Due to the limitation of our clinical scanner, we have used the maximum 12 directions of the diffusion gradient that was allowed. As for the maximum b-value of 2000 s/mm^2^, increasing it would have lower down our signal to noise ratio as our rabbit brain is comparatively smaller than human brain.

In future, we definitely look forward to carry out GQI protocol through scanners that are capable of providing higher b-values and more diffusion gradient directions. There is always a challenging issue of optimizing the b-table for diffusion scans as the performance of the b-table depends largely on the reconstruction method, and the best table for one method may not work for another. More studies are needed to resolve this question. As in our study here, we believed that we have taken the best from our clinical scanner, both time wise and signal wise.

## Conclusions

Our results demonstrated that the white matter anisotropy and R2 values increased with age. Regional brain DTI tractography of all region of interest (ROI) revealed refinement in fiber pathway architecture with maturation. Furthermore, GQI indices revealed that the major white tract as well as the gray matter change during the mature period and may represent important targets for in vivo human studies. Therefore, this developing rabbit brain DTI and GQI database and techniques could be used for educational purposes and neuroscience investigations.
